# Dietary Intake and Food Habits of Pregnant Women Residing in Urban and Rural Areas of Deyang City, Sichuan Province, China

**DOI:** 10.3390/nu5082933

**Published:** 2013-07-31

**Authors:** Haoyue Gao, Caroline K. Stiller, Veronika Scherbaum, Hans Konrad Biesalski, Qi Wang, Elizabeth Hormann, Anne C. Bellows

**Affiliations:** 1 Institute of Social Sciences in Agriculture, Gender and Nutrition (430b), University of Hohenheim, Garbenstraße 30, Stuttgart 70599, Germany; E-Mails: corkiagao@msn.com (H.G.); caroline-stiller@gmx.de (C.K.S.); 2 Institute of Biological Chemistry and Nutrition (140a), University of Hohenheim, Garbenstraße 30, Stuttgart 70599, Germany; E-Mail: biesal@uni-hohenheim.de; 3 Medical Society of Deyang City, Sichuan, Public Health Bureau of Deyang, Lushan Nan Road No.299, Jingyang District of Deyang City, Deyang 618000, China; E-Mail: wangqi8311@163.com; 4 Europäisches Institut für Stillen und Laktation, Wittberg 14, Kramsach 6233, Austria; E-Mail: elizhorman@aol.com; 5 Department of Public Health, Food Studies, and Nutrition, David B Falk College, Syracuse University, Syracuse, NY 13244, USA; E-Mail: acbellow@syr.edu

**Keywords:** food habits, 24-h dietary recall, pregnancy, urban, rural, China

## Abstract

Micronutrient deficiencies and imbalanced dietary intake tend to occur during the reproductive period among women in China. In accordance with traditional Chinese culture, pregnant women are commonly advised to follow a specific set of dietary precautions. The purpose of this study was to assess dietary intake data and identify risk factors for nutritional inadequacy in pregnant women from urban and rural areas of Deyang region, Sichuan province of China. Cross-sectional sampling was applied in two urban hospitals and five rural clinics (randomly selected) in Deyang region. Between July and October 2010, a total of 203 pregnant women in the third trimester, aged 19–42 years, were recruited on the basis of informed consent during antenatal clinic sessions. Semi-structured interviews on background information and 24-h dietary recalls were conducted. On the basis of self-reported height and pre-pregnancy weight, 68.7% of the women had a pre-pregnancy body mass index (BMI) within the normal range (18.5 ≤ BMI < 25), 26.3% were found to be underweight with a BMI <18.5 (20.8% in urban *vs*. 35.6% in rural areas), while only 5.1% were overweight with a BMI ≥30. In view of acceptable macronutrient distribution ranges (AMDRs) the women’s overall dietary energy originated excessively from fat (39%), was low in carbohydrates (49.6%), and reached the lower limits for protein (12.1%). Compared to rural areas, women living in urban areas had significantly higher reference nutrient intake (RNI) fulfillment levels for energy (106.1% *vs.* 93.4%), fat (146.6% *vs.* 119.7%), protein (86.9% *vs.* 71.6%), vitamin A (94.3% *vs.* 65.2%), Zn (70.9% *vs.* 61.8%), Fe (56.3% *vs.* 48%), Ca (55.1% *vs.* 41%) and riboflavin (74.7% *vs.* 60%). The likelihood of pregnant women following traditional food recommendations, such as avoiding rabbit meat, beef and lamb, was higher in rural (80%) than in urban (65.1%) areas. In conclusion, culturally sensitive nutrition education sessions are necessary for both urban and rural women. The prevalence of underweight before conception and an insufficient supply of important micronutrients were more pronounced in rural areas. Therefore, attention must be given to the nutritional status, especially of rural women before, or at the latest, during pregnancy.

## 1. Introduction

Since 2006, China had entered the fourth wave of a baby boom period with an annual increase of about 20 million babies [1]. Research at the international level shows that maternal malnutrition is linked to fetal programming for adult diseases and increases the risk of pregnancy-associated complications [2,3,4,5,6]. In China, maternal anemia (Hb < 110 g/L) (14.5%) [7], nutritional deficiencies of Ca, Fe, Zn, vitamin A, folic acid and protein as well as low energy intake are common [8,9,10,11,12]. Based on the results of a study conducted in Chengdu, the capital of Sichuan province, the prevalence of anemia among pregnant women in the third trimester was 35.0% [13]. However, instead of the internationally accepted anemia cutoff point for pregnant women of Hb < 110 g/L [14], a lower threshold of Hb < 100 g/L was applied by Zhou *et al.* [13]*.* In one study, which included 292,568 women in three provinces of China, the prevalence of low body mass index (BMI) before conceiving was 21.6% (BMI < 18.5, urban 24.9% *vs.* rural 21.0%) [15]. Both pre-pregnancy underweight and maternal anemia are known to be associated with an increased incidence of low-birth-weight (LBW: <2.5 kg) and prematurity [15,16]. In eight provinces of China, the prevalence of premature birth (28–37 weeks), LBW, and high birth weight (>4 kg) of neonates was 9.6%, 3.9%, and 9.2%, respectively, with higher proportions of prematurity (13.1% *vs.* 8.4%) and LBW (8.1% *vs.* 2.4%) in urban than in rural areas. On the other hand, slightly more babies with high birth weights were found in rural than in urban regions (10.0% *vs.* 7.1%) [17].

For more than a decade, China has been facing the “double burden” of under- and over-nutrition [18,19]. The Chinese diet became proportionately rich in fat, as the dietary energy from fat increased from 22% in 1989 to 31% in 2000. In 2000, 44.4% of the Chinese population aged 20–59 had a fat intake exceeding 30% of the total energy intake [18]. Regarding food intake, rural residents in China appeared to consume more grains and fewer animal-based foods than urban residents [20], which is well known to be associated with a lower energy and macro- as well as micro-nutrient intake [21,22]. Furthermore, in accordance with traditional Chinese culture, women are expected to follow a set of dietary precautions during the childbearing period [23,24,25]. Research indicates that most of these traditional food taboos have no scientific justification or are even harmful to women’s health [25]. On this basis, the objectives of this study were to provide baseline data on dietary intake and food habits in order to identify the risk of nutritional inadequacy, and to give appropriate dietary recommendations for pregnant women in urban and rural areas of Deyang City, Sichuan province, China.

## 2. Participants and Methods

### 2.1. Study Design and Population

**Figure 1:**
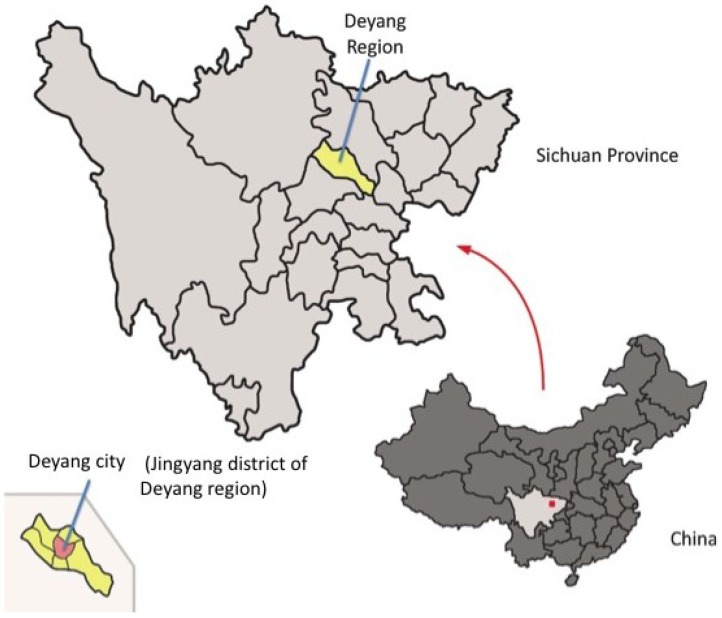
Map of study area.

Since, on a national level, the vast majority (96%) of Chinese women delivered in medical institutions during 2006–2010 [26], a cross-sectional study design was applied in medical institutions. Two of five hospitals in the urban part of Deyang City (Jingyang District, see Figure 1) and five of 12 clinics in rural areas were randomly selected. Between July and October 2010, a total of 203 pregnant women in the third trimester, aged 19–42, were recruited with informed consent during antenatal clinic sessions. Finally, 201 pregnant women were analyzed (62.7% in urban, 37.3% in rural areas); two were excluded because their residences were missing. A semi-structured interview on background information and a 24-h dietary recall were conducted directly in the clinic and/or during a home visit, depending on the time availability of the pregnant women.

### 2.2. Dietary Assessment

To characterize the average usual dietary intake of pregnant women in the study area, the 24-h dietary recall method was applied. Women were prompted to recall all food items including snacks, extraordinary beverages and shared dishes consumed the preceding day and were advised to describe their food intake according to daily routine. To help the respondents estimate portion sizes, measuring aids were shown to the women: two self-painted templates illustrating standardized sizes of Chinese dishware and rough suggestions of portion sizes of food consumed; household measures were also used, including a set of spoons and a measuring cup to quantify smaller portion sizes and beverage volume, respectively. At the end of the recall, items noted down were read to the respondent in order to guarantee completion of the protocol. Traditional food beliefs and taboos were defined on the basis of the knowledge of medical staff and published evidence [25,27]. Specific questions on nutrient supplementation were not asked.

### 2.3. Anthropometric Assessment

For a rough estimate of nutritional status, anthropometric data including height, pre-pregnancy weight, mid-upper arm circumference (MUAC), and weight in late pregnancy were assessed. Except for MUAC, the women themselves provided all the data. Currently, there is no international consensus on the MUAC cutoffs for the detection of women with poor nutritional status, who are simultaneously at risk for adverse birth outcomes. On the basis of several studies [28,29,30,31,32,33] and due to the need for international comparison, we chose a MUAC value of 23 cm as the cutoff point. Since the WHO collaborative study [34] was clearly relevant to the Chinese population, proposing a MUAC <22 cm as a proxy measure for poor maternal nutritional status, this cutoff point is also referred to in this study. This allows a more pictorial representation of the sample and improves comparison, as this cutoff is cited by a range of studies [3,35,36]. In reference to WHO BMI standards [37], the health status of women before pregnancy was defined as underweight (BMI < 18.5), normal (18.5 ≤ BMI < 25), overweight (BMI ≥ 25), or obese (BMI ≥ 30).

### 2.4. Statistical Methods and Food Intake Analyses

Statistical data analyses were performed using the SPSS 19.0 software package (IBM, New York, NY, USA). Significant differences between urban and rural areas were determined at *p* ≤ 0.05. The 24-h recall records were converted to nutrient intakes using the software NutriSurvey. Preparatory work included the adjustment of food databases for Chinese conditions, the definition of standard food recipes based on a comparison of several Sichuan cuisine books, and the conversion of recalled volumes into weights.

Adequacy of nutrient consumption was evaluated by applying the Chinese dietary reference intakes (DRIs) with light physical activity level (PAL) [38] adopted by the Institute of Nutrition and Food Safety China (INFSC) [39,40]. Reference values were recommended nutrient intake (RNI) or adequate intake (AI) for pregnant women. To better judge the need for nutritional assistance, insufficient nutrient intake was determined as below 80% of the RNIs or AIs respectively, while excessive intake of energy or fat was set above 120% of reference. The rationale for using this 20% allowance was that the DRIs are designed with a risk allowance, which better satisfies individual physiological fluctuations of nutrient needs. Thus, the failure to attain the reference intakes may not be equated with falling below the nutrient requirements, as a reference value is not a strict threshold [41].

### 2.5. Ethical Clearance

The study conformed to the provisions of the 1995 Declaration of Helsinki (revised in Edinburgh 2000) and was approved by the Medical Committee of Deyang City, China. The purpose of the study was explained to pregnant/lactating women and parents of eligible children, who gave informed consent.

## 3. Results

### 3.1. Socio-Demographic Characteristics

Most respondents were Han Chinese (97%), primiparae (85.4%), had completed at least primary education (95.5%), and contributed to their families’ income (74.6%). Before conceiving, most were employed in the service sector (36.7%), about one fifth were homemakers (19.1%), and a similar number were employed in skilled or professional labor (19.7%). Others reported having been self-employed (14.9%) or engaged in unskilled or manual work (9.6%). Compared to rural areas, a higher proportion of women from urban areas had a college degree (39.2% *vs.* 8.2%), were self-employed (19.8% *vs.* 6%) or engaged in skilled or professional labor requiring post-secondary degrees (24.7% *vs.* 10.5%), and contributed a higher percentage to the total household income. A higher proportion of women from the rural sites experienced unemployment (22.4% *vs.* 17.4%) and exceeded the employment rates of their urban counterparts in the service sector (44.8% *vs.* 32.2%) and the unskilled labor force (16.4% *vs.* 5.8%) before pregnancy.

Including the expected child, rural women appeared on average to have significantly (*p* = 0.009) more children (1.28 ± 0.53) at a significantly (*p* < 0.001) earlier age (24.1 ± 4.65 years) than urbanites, at an average of 26.5 ± 3.89 years and with 1.09 ± 0.29 children. In line with the one-child family policy, the clear majority of urban women were primiparae (90.8%) compared to rural subjects (75.5%). Third pregnancies occurred only in rural areas (3.8%).

On the basis of self-reported height and pre-pregnancy weight, most women had a pre-pregnancy BMI within the normal range before conceiving (68.7%; 18.5 ≤ BMI < 25). About a quarter were found to be underweight with a BMI of <18.5 (26.3%; 20.8% in urban *vs.* 35.6% in rural areas); while a small number were overweight with a BMI of ≥25 to <30 (5.1%). None of the women had been obese (BMI ≥ 30) before conceiving. During the third trimester, the mean value of MUAC was 25.8 ± 2.9 cm, showing no significant (*p* = 0.130) difference between women from urban (26.1 ± 2.7 cm) and rural (25.4 ± 3.3 cm) areas. When applying 22 cm as the cutoff point for MUAC, 7.3% of pregnant women were found to be malnourished (6.7% *vs.* 8.3% in urban and rural areas, respectively). Considering a MUAC of 23 cm as threshold, the number of women with poor nutritional status increased to 14.7%, (11.8% *vs.* 19.4% in urban and rural areas, respectively).

### 3.2. Antenatal Food Habits and the Cultural Beliefs and Fears Entailed

The most important sources from which participants heard about traditional food culture were the family (63.5%), elders (37.2%), and non-family including friends and neighbors (35.8%). Books and booklets played a minor role in transmitting knowledge about traditional rituals or taboos (6.6%).

Seventy point six percent (70.6%) of participants adhered to specific food taboos during their pregnancies while 29.4% did not restrict their eating. The different reasons for these food precautions were qualitatively collected (see Table 1).

**Table 1:** Stated reasons for practicing special dietary precautions during pregnancy among women who adhered to any food taboos (*n* = 142).

Food taboos during the pregnancy & qualitative analysis of cultural fears entailed			
	Urban *n* = 82	Rural *n* = 60	
*Metaphysically “cold” foods*	*n* (%)	*n* (%)	
Crab	0	1 (1.7)	May induce premature birth or miscarriage
Soft-shelled turtle	0	1 (1.7)	
Snake	1 (1.2)	0	
Ice cream	0	1 (1.7)	
Sprite	0	1 (1.7)	
“Cool” foods	4 (4.9)	8 (13.3)	
*Metaphysically “hot” foods*	*n* (%)	*n* (%)	
Dog	1 (1.2)	2 (3.3)	Dog flesh is hot in the hot-cold classification of foods. The baby may bite the mother
*Metaphysically “toxic” foods*	*n* (%)	*n* (%)	
Rabbit	58 (70.7)	40 (66.7)	Causes harelip or cleft palates in the fetus, rabbit head
Eel	8 (9.8)	10 (16.7)	Causes foaming at the mouth of the newborn
Beef	20 (24.4)	22 (36.7)	Child may have a stubborn temper like an ox
Fish	1 (1.2)	2 (3.3)	Baby may get bacterial infection of the skin more easily
Duck	7 (8.5)	9 (15)	Newborn’s neck will be weak or lack power. Causes duck head
Lamb, mutton, goat	62 (75.6)	49 (81.7)	Fetus will suffer from epilepsy
Sow	1 (1.2)	1 (1.7)	
Chinese anise	1 (1.2)	0	Harmful due to lots of flavors, may contain rosin (colophony), not fresh enough, traditional reasons
Clove	1 (1.2)	0	
Hot pot	1 (1.2)	0	
Braised/stewed meat	4 (4.9)	0	
Bacon	1 (1.2)	0	
Leek	1 (1.2)	0	
Sauerkraut	1 (1.2)	0	

Modified from Lee *et al.* 2009 [25].

Eighty percent (80%) of rural women practiced some food avoidance, which was a markedly higher percentage compared to 65.1% in the urban setting. Among the women adhering to food taboos during pregnancy, the most commonly cited food items were lamb, mutton, goat (78.2%) followed by rabbit (69%), beef (29.6%), eel (12.7%), duck (11.3%), foods with a “too cold” property (8.5%), or fish (2.1%). Spicy foods (e.g., hot pot) and foods with multiple flavors (e.g., clove, braised/stewed meat) were avoided by 7% for food purity and perceived safety reasons.

Half (54.7%) of the women interviewed increased their consumption of some types of foods during pregnancy. A higher percentage of urban subjects (60.3%) than rural subjects (45.3%) said they had increased consumption of any foods. Soup with varieties of meat and vegetables accounted for 85.5% of all food increases. Three subjects indicated that they were eager to consume “as much soup as possible” for the well being of both mother and child. On the whole, soup with Chinese olives and pig stomach (*n* = 30), salsola grass and meat (*n* = 18), and green beans and pig intestine (*n* = 15), were the most commonly mentioned types of soup, with these ingredients appearing in a large number of other soups cited as well.

The concept of “reducing internal heat of the fetus” was by far the most frequently stated reason for almost all types of food increases. (In traditional Chinese medicine, the state of health is seen as a balance between *yin* and *yang*, or *cold* and *hot*. Illness is viewed as an imbalance between these two opposed forces. Classification according to *yin* and *yang* applies to many things such as stages of life, foods, and herbs. To assure one’s vital energy (ch’i) [42] the “right” foods have to be selected according to the particular life stage or health condition of the individual [43]. Cold and hot are defined by type of food, thereby the terms *yin* and *yang* suggest “the inherent nature of the food”, not its temperature [44]. Pregnancy is believed to cause a harmful disequilibrium of *yin* and *yang* in the female body [45]. During the first three months of pregnancy, a woman is believed to be feeble, cold, and lacking in vigor. In the subsequent three months she is in a neutral condition and in the final three months she is hot and tonic. Hence, during second trimester a woman starts eating cold foods to better counteract the hot condition that may arise in the final stage of pregnancy [46].) Other reasons included “nutritious” (fish soup, *n* = 4), “improves breast milk flow” (soup with crucian carp, *n* = 3), “improves birth weight” (soup with cuttlefish, *n* = 2), “good for baby’s stomach” (soup with Chinese redbud flower and pig stomach, *n* = 1), “prevents nausea” (water with towel gourd, *n* = 1), and “promotes baby’s intelligence” (walnut, *n* = 1). The most mentioned non-soup food items were boiled or fried goose eggs (*n* = 9), assumed “to avoid boils in the newborn”. The consumption of duck eggs (*n* = 3) was believed to “reduce fever”.

### 3.3. Result of 24-h Recall

#### 3.3.1. Dietary Intake of Participants at the Two Survey Sites (Urban *vs.* Rural)

On average, 12.1%, 39.0%, and 49.6% of dietary energy originated from protein, fat, and carbohydrates, respectively. The contribution of fat to total energy was higher among urban mothers, though the opposite was the case with respect to the proportion of energy from carbohydrates. The percentage of energy derived from protein did not differ significantly between the two survey sites (see Table 2).

**Table 2:** Energy derived from protein, fat and carbohydrates expressed as the percentage of the total energy intake (kcal) of pregnant women living in urban and rural areas.

% of energy	AMDRs ^•^	Total (*n* = 192)	Urban (*n* = 125)	Rural (*n* = 67)	*p*-Value ^1^
Derived from protein	10–15	12.1 ± 2.9	12.3 ± 2.8	11.6 ± 3	0.118 ns
Derived from fat	20–30	39 ± 11.2	40.4 ± 10.8	36.6 ± 11.6	0.025 *
Derived from carbohydrate	55–65	49.6 ± 11.4	48.1 ± 11.1	52.4 ± 11.7	0.012 *

^•^ Acceptable macronutrient distribution ranges (AMDRs) adopted by the INFSC [39,40]; ^1^ Significance level; ns, *p* > 0.05 non-significant; * *p* ≤ 0.05 significant.

Intakes of energy and selected nutrients are summarized in Table 3. Analyses of urban-rural differences revealed not only significant differences in the intake of energy but also in protein, fat, vitamin A, riboflavin, Ca, Fe, and Zn. In general, urban women invariably exceeded mean values of the rural women. Yet, at both sites, average intake of thiamin, riboflavin, Ca, Mg, Fe, and Zn was below 80% of RNI. Energy consumption fell into a desirable range close to RNI fulfillment, protein intake in urban areas was slightly above whilst the rural share fell short of 80% of RNI. With respect to judging the risk of chronic disease, the intake of fat clearly exceeded the upper limit of the self-calculated reference range, whilst the intake of carbohydrates fell below the lower limit of the healthy range.

**Table 3:** Energy and nutrient intakes of rural and urban pregnant women in Deyang city in relation to fulfillment of the national recommended nutrient intakes (RNIs) (adopted by the INFSC 2002/2004).

		Total (*n* = 192)			Urban (*n* = 125)			Rural (*n* = 67)			
Energy and nutrient (unit/day)	RNIs/AIs ^•^	Mean ± SD	% fulfillment RNI	Range	Mean ± SD	% fulfillment RNI	Range	Mean ± SD	% fulfillment RNI	Range	*p*-Value ^1^ Mean ± SD
Energy (kcal)	2300	2338 ± 844	101.7	566–5705	2441 ± 834	106.1	767–5705	2148 ± 837	93.4	565–4514	0.021 *
Protein (g)	85	69.4 ± 26.7	81.6	15.3–171.2	73.9 ± 26.9	86.9	16.7–171.2	60.9 ± 24.4	71.6	15.3–130.6	0.001 ***
Fat (g)	51–77 ^†^	105.7 ± 58	137.3 ^‡^	11.3–348.1	112.9 ± 57.8	146.6 ^‡^	11.3–348.1	92.2 ± 56.4	119.7 ^‡^	14.8–282.1	0.018 *
Carbohydrate (g)	316–374 ^†^	281.5 ± 103.9	89.1 ^§^	89.1–670.6	286.5 ± 102.5	90.7 ^§^	124.2–616.6	272.1 ± 106.6	86.1 ^§^	89.1–670.6	0.311 ns
Vitamin A (µg RE)	900	757.5 (±*920.8*)	84.2	10–8520.7	849.1 (±*1032*)	94.3	46.1–8520.7	586.4 (±*638.1*)	65.2	10–3093	0.005 **
Vitamin C (mg)	130	104.3 (±*110.1*)	80.2	1.6–744.1	106.8 (±*112.4*)	82.2	1.6–744.1	99.7 (±*106.4*)	76.7	7.28–697.8	0.589 ns
Thiamin (B1) (mg)	1.5	0.81 ± 0.41	54	0.25–2.66	0.81 ± 0.39	54	0.25–2.53	0.79 ± 0.44	52.7	0.26–2.66	0.517 ns
Riboflavin (B2) (mg)	1.7	1.19 ± 0.58	70	0.16–4.26	1.27 ± 0.58	74.7	0.16–4.26	1.02 ± 0.53	60	0.17–2.84	0.003 **
Ca (mg)	1200	602.1 ± 378.3	50.2	33.5–2788.4	660.8 ± 379.6	55.1	81.3–2788.4	492.5 ± 353	41	33.5–1530.9	<0.001 ***
Mg (mg)	400	300.4 ± 164.5	75.1	77.9–1202.8	311.5 ± 160.9	77.9	97.5–1127.6	279.8 ± 170.4	70	77.9–1202.8	0.069 ns
Fe (mg)	35	18.7 ± 9.8	53.4	5.2–88.9	19.7 ± 10.4	56.3	5.2–88.9	16.8 ± 8.3	48	6.2–58.2	0.019 *
Zn (mg)	16.5	11.2 ± 4.7	67.9	3.5–32.6	11.7 ± 4.8	70.9	4.3–32.6	10.2 ± 4.5	61.8	3.5–28	0.025 *

^•^ Recommended nutrient intakes (RNIs) and adequate intakes (AIs)—adopted by the INFSC [39,40]; ^†^ As RNI values are confined to protein and micronutrients, we judged the average intake of fat and carbohydrates by referring to self-calculated healthy ranges in g/day on the basis of the AMDRs, defined as: “a range of intake for a particular energy source (protein, fat, or carbohydrate), expressed as a percentage of total energy (kcal), that is associated with reduced risk of chronic disease while providing adequate intakes of essential nutrients” [47]; calculated from total energy in kcal, using the energy yield of macronutrients: carbohydrate = 4 kcal/g; protein = 4 kcal/g; fat = 9 kcal/g [48,49], assuming the AMDRs: fat (20%–30% of energy), carbohydrates (55%–65% of energy) [39,40]; ^‡^ percentage relates to the upper limit of the self-calculated reference range; ^§^ percentage relates to the lower limit of the self-calculated reference range; ^1^ Significance level; ns, *p* > 0.05 non-significant; * *p* ≤ 0.05 significant; ** *p* ≤ 0.01 very significant; *** *p* ≤ 0.001 highly significant.

#### 3.3.2. Food Groups and Sources of Energy and Nutrients

Rice was the most popular staple food in the total study population with a daily consumption of 178.4 g/day, which was markedly higher than the consumption of other grains (57.5 g/day) (see Table 4).

**Table 4:** Average food consumption of pregnant women living in urban and rural areas according to selected food groups.

Amount (g/day)	In total	Urban	Rural	*p*-Value ^1^
	(*n* = 192)	(*n* = 125)	(*n* = 67)	
Rice/its products	178.4 ± 94.5	176.6 ± 105.1	181.9 ± 71.2	0.101 ns
Wheat and other grains/their products	57.5 ± 133.5	57.6 ± 93.2	57.4 ± 187.8	0.053 ns
Starchy tubers, roots	30.6 ± 63.2	32.4 ± 66.8	27.3 ± 56.1	0.911 ns
Soy products	56.1 ± 138.4	65.3 ± 151.6	38.9 ± 108.6	0.459 ns
Soya sauce and bean paste	6.9 ± 6.2	7.4 ± 6.4	6.0 ± 5.7	0.123 ns
Vegetables	164.7 ± 170.4	183.7 ± 186.9	129.3 ± 128.4	0.042 *
Preserved vegetables	11.7 ± 52.4	15.6 ± 64.1	4.4 ± 12.2	0.083 ns
Fruits	517.1 ± 626.3	498.6 ± 619.2	551.6 ± 642.6	0.332 ns
Nuts and seeds	26.8 ± 52.0	29.0 ± 51.8	22.6 ± 52.6	0.202 ns
Pork	100.8 ± 105.1	100.7 ± 96.5	101.0 ± 120.4	0.567 ns
Other meats/poultry/offal	37.0 ± 58.8	41.0 ± 62.7	29.5 ± 50.4	0.120 ns
Cow’s milk	147.3 ± 196.3	172.9 ± 203.9	99.5 ± 172.7	0.012 *
Chicken eggs	55.0 ± 58.1	51.5 ± 53.6	61.7 ± 65.5	0.624 ns
Seafood	16.8 ± 42.9	22.4 ± 50.2	6.4 ± 20.8	0.006 **
Vegetable oil	44.4 ± 37.0	49.2 ± 39.2	35.5 ± 30.7	0.016 *
Animal fats	1.2 ± 4.8	1.6 ± 5.8	0.3 ± 1.9	0.026 *

^1^ Significance level; ns, *p* > 0.05 non-significant; * *p* ≤ 0.05 significant; ** *p* ≤ 0.01 very significant.

Significant differences in food group consumption disclosed in the categories of cow’s milk and seafood. Further, average consumption of vegetables and visible oils and fat was invariably found to be significantly higher for the urban site. There was a slight trend suggesting the share of animal-based foods to be higher among urban women. This assumption is substantiated when comparing average animal-based food consumption, including animal fats, meats, poultry, offal, seafood and secondary products such as chicken eggs, milk, or dairy products at the urban (403.6 ± 252.9 g/day) and rural sites (305.8 ± 220.8 g/day) in the Deyang area (*p* = 0.008 **). The daily intake of plant-based food was comparable (*p* = 0.326 ns) in rural (1049.1 ± 716.5 g/day) and urban areas (1107.9 ± 714.7 g/day).

The contribution of different food sources to the overall dietary intake was another focus of the study. Fats, oils, and lard contributed 17.5% to total energy and made up nearly half (42.8%) of the total dietary fat. Animal-based foods (50.2%, 35.2 g) and foods from plant sources (44.8%, 31.4 g) made a balanced contribution to total protein. By contrast, the basic source of vitamin A was plant-based (62.7%) as compared to 34.2% derived from animal-based foods, equaling 475.5 µg and 259.5 µg of total intake, respectively. A similar pattern applied to the overall Fe and Zn intake with 71.4% (13.5 mg) and 53.8% (6.4 mg) respectively originating from plant foods as compared to merely 20.4% (3.9 mg) and 30.8% (3.7 mg) originating from animal foods. With respect to the total Ca, 35.4% (224.4 mg) was provided by plant-based foods and 45.3% (287.4 mg) was derived from animal-based foods. Milk and dairy products contributed the most to the overall Ca intake, with 36.2% (229.7 mg).

Furthermore, in this study, dietary diversity was generally found to be higher among urban women. This conclusion is drawn from the finding that snacking times, the total number of snacks, as well as the number of different dishes with various ingredients consumed at one sitting tended to be higher among urban women. Among rural women, repeatedly serving the same dish or leftovers was more common. In total, for the urban area about 263 different types of food items have been recorded during evaluation of the 24-h recall, as compared to 200 different types of foods for rural women which equals a difference in variety of 24%.

## 4. Discussion

### 4.1. Food Taboos and Special Foods Consumed during Pregnancy in Rural and Urban Areas

With respect to taboos among Hong Kong Chinese women, avoidance of snake (92%), iced foods (83%), or beef (40%) is still common during pregnancy. Lee *et al.* [25] point to the danger of nutritional deficiencies being caused or aggravated by overzealous adherence to dietary prescriptions. In our results, it appears that traditional dietary practices did not negatively affect maternal nutritional status. This was based on two findings. First, there were no significant differences in nutrient intake among those practicing any food taboos or increasing consumption of special foods, and those who did not change their eating behavior during pregnancy. Second, the great dietary diversity in Chinese cuisine provides adequate alternatives to meet a balanced diet. However, beef or fish (including eel), which were avoided by some women, are widely considered to be healthy and nutritious food choices, hence it may be desirable to encourage women to include these items in their diet. Furthermore, the socio-moral pressures that pregnant women face from non- or partial adherence to food taboos should not be overlooked. In the study of Lee *et al.* [25], family (65%) and friends (22%) were listed as the most important channels for imparting antenatal taboos, which is consistent with outcomes of this study. The predominant role of the family in promoting cultural practices implies that various expectations are imposed upon pregnant women, making the psycho-cultural experience of pregnancy even more complicated. As a consequence, in the event of miscarriage or fetal ill-health, women might be held accountable by their relatives [25]. This suggests the need to clarify that the principles behind taboos are based on folklore rather than on scientific evidence.

With respect to differences between rural and urban areas, more rural than urban women practiced food avoidance, while more urban than rural women increased their consumption of foods. An explanation for this could be that rural women were more influenced by traditional culture, while urban women had a higher educational level and more access to nutritional knowledge.

### 4.2. Dietary and Food Intake Patterns

Evaluation of the 24-h recalls indicated the need to improve the dietary intake of pregnant women living in Sichuan province, although the overall picture showed some satisfactory dietary habits as well.

#### 4.2.1. Dietary Intake in Rural and Urban Areas

Findings of this study clearly show that there are distinct differences in energy as well as nutrient intakes among pregnant women residing in urban and rural areas of Deyang city (Table 3). In general, average energy consumption met the range of 20% allowance of RNI. Referring to the macronutrient composition of pregnant women’s diets, the consumption of carbohydrates could be increased to meet the lower limit of the self-calculated healthy range. Protein intake was deficient in the rural areas, whilst urbanites were still above 80% of RNI. The assumed fat intake was excessive at both survey sites, exceeding the upper healthy range. The dietary intake of most micronutrients was more or less deficient in both study sites but low intake was more pronounced in rural than in urban areas. The observed trend of higher intakes among urban mothers is not holistically reflected by the 2002 NNHS of pregnant women [50] but is, however, widely mirrored by data of the 1992 and 2002 NNHS including individuals of all ages [20] and is clearly evident in the data on adult women in Jilin province [21]. In summary, apart from fat intake, urban women had more nutritious and diversified diets than rural women in this study.

Taking these factors together, it can be reasonably assumed that rural women are more vulnerable to nutritional imbalances than are urban women.

#### 4.2.2. Food Group Consumption in Rural and Urban Area

As indicated in Table 4, urban women consumed more animal-based foods compared to rural women. Inferentially, rural women could increase animal foods to improve their dietary quality. Normally, both rural and urban women bought food from local traditional markets. Food diversity is much higher in urban than in rural markets and the markets are much closer to home for urban families than for rural ones. High price and low availability in rural markets could be the reason that rural women consumed less cow’s milk and seafood than urban women (Table 4). Low food diversity in rural markets could also be responsible for lower vegetable consumption by rural women. Furthermore, rural women tended to eat home-grown vegetables of which there were limited varieties, which is assumed to contribute to the insufficient representation of vegetables in rural women’s diets.

#### 4.2.3. Comparison of Dietary Patterns with Related Studies

With respect to the daily reference ranges suggested by Yang [51], women in the Deyang study sample had a better supply of animal-based foods than the pregnant women in the 2002 NNHS study [50] (see Table 5). Furthermore, fruits seemingly enjoyed great popularity as an integral part of the daily diet, although the very high fruit consumption in the Deyang study sample (Table 5 and Table 6) must be seen in relation to the widespread consumption of watermelon during the study period that may have boosted the average weight. Intakes of staples and meat products meet the recommended range, but scatter at the lower limit, thus indicating a possible area where nutrient intake could be improved. The category of milk and dairy products (159 g/day), though being much more satisfactory compared to 19 g/day in the 2002 NNHS, could be boosted still further (Table 5). Of special concern is the excessive addition of cooking oil during food preparation, which results in passive overconsumption of fat. In theory, high fat content not only increases the energy content but, first and foremost, negatively affects protein and micronutrient density [52,53]. In summary, when compared with participants of the 2002 NNHS [54], participants in the Deyang study sample appeared to consume considerably more livestock products and cooking oils, and far fewer staples and vegetables. Except for oil intake, the contribution of all other food categories to the total diet should be increased, especially in women with a low BMI.

**Table 5:** Average amount of selected food groups consumed by pregnant women (*n* = 310) in the 2002 NNHS [50] compared with consumption patterns of participants of our study (*n* = 192) and recommendations by Yang, 2008 [51].

Food group (g/day)	2002 NNHS National level China Pregnant women (*n* = 310) (whole pregnancy period)	Deyang, 2010 Urban and rural areas Pregnant women (*n* = 192) (3rd trimester)				Recommendation (Yang [51], 2008)
		In Total (*n* = 192)	Urban (*n* = 125)	Rural (*n* = 67)	*p*-Value ^1^	
Staples	461	266.6 ± 179.4	266.6 ± 159.4	266.6 ± 213.1	0.882 ns	250–400
Soy products	13	63.0 ± 138.6	72.7 ± 151.8	44.9 ± 108.5	0.114 ns	30–50
Vegetables	285	176.4 ± 174.1	199.3 ± 190.8	133.8 ± 128.1	0.010 **	300–340
Fruits	81	517.1 ± 626.3	498.6 ± 619.2	551.6 ± 642.6	0.332 ns	200–400
Meats, fish	96	154.6 ± 123.9	164.0 ± 124.3	137.0 ± 122.2	0.061 ns	125–225
Milk, dairy products	19	158.7 ± 198.7	186.5 ± 207.0	106.9 ± 172.0	0.006 **	300
Chicken eggs	25	55.0 ± 58.1	51.5 ± 53.6	61.7 ± 65.5	0.624 ns	- **^•^**
Vegetable oil	31	44.4 ± 37.0	49.2 ± 39.2	35.5 ± 30.7	0.016 *	25

^•^ No recommendation provided; ^1^ Significance level; ns, *p* > 0.05 non-significant; * *p* ≤ 0.05 significant; ** *p* ≤ 0.01 very significant.

**Table 6:** Average food consumption by the Chinese population in 1982, 1992, and 2002 [20], compared with food groups consumed by pregnant women in Deyang study.

	NNHS National level China Covering individuals of all ages			Deyang, 2010 Urban and rural areas Pregnant women (3rd trimester)
*Food group (g/day)*	*1982*	*1992*	*2002*	*2010*
Grains/its products	510	440	402	236
Vegetable & fruit	368	369	331	724
Animal sources	61	117	160	368
Visible oil and fat	18	30	41	46

For the period 1982 to 2002, Table 6 clearly demonstrates the general trend toward an energy-dense diet that is low in carbohydrates. Altogether, the share of staples such as rice, cereals, or potatoes is declining while the share of animal-based foods and vegetable oils is rising. Although the data of this study smoothly integrate into the overall image of a continuing re-structuring of dietary patterns, it is important to keep the limitations in mind when comparing a representative national sample with a much smaller local sample.

### 4.3. Recommendations to Improve Dietary Quality

#### 4.3.1. Improving Nutrient Density of the Diet in Rural and Urban Areas

High fat intake was common in both urban and rural areas equaling 146.6% and 119.7% of the upper desirable limit (Table 3). The percentage of energy derived from fat shown in the Deyang study (39%) is quite alarming, though it is not reflected in the BMI distribution of Deyang respondents. From the mid-1960s onwards, a steady increase in dietary energy worldwide took place [19,55]. Fried dishes (fried-fragrant, stir-fried, or deep-fried) are very popular in Sichuan province and are, thus, firmly established in the daily diet. As expected, in this study vegetable oils alone provided 41.7% of total fat consumed and constitute about half (17.1%) of the recommended maximum percentage of total energy that should be derived from dietary fat (30%) [39,40]. Types of oils consumed were rapeseed oil (93.3%), sesame seed, chili, or salad oil (6.7%). A desirable diet comprises saturated (SFA), monounsaturated (MUFA), and polyunsaturated (linoleic acid—LA; α-linolenic acid—ALA) fats in the ratio of 35%, 44%, and 23%, respectively [56]. Hence rapeseed oil has a good fatty acid composition to complement a well-balanced diet (SFA 8%, MUFA 64%, LA 23%, ALA10%) [57]. Nevertheless with the unsatisfactory nutrient density we found, it is critically important to encourage women to limit the usage of oil in cooking or, as a second approach, to give priority to non-fried (e.g., red-braised) over fried dishes. Then, nutrient density would be much more satisfactory given that Chinese cuisine also includes a considerable variety of nutrient-dense (meat, grains) and energy-dilute foods (vegetables, legumes, fruits), despite a relatively high percentage of energy as fat from added oils. Thus, small behavioral changes could result in major nutritional improvements. In addition, to further boost nutritional status, it is strongly recommended that the consumption of nutrient-dense foods be increased, while simultaneously curbing oil consumption. This issue is of particular concern, given that the energy consumption shown in our study is not excessive. Both sites are at risk of falling substantially short of the energy reference value unless the reduction in dietary fat is accompanied by behavioral changes. Furthermore, the nutrient gap with respect to RNIs will remain decisive. Hence, the selection of nutrient-dense foods, accompanied by a restricted usage of cooking oil, is the first order of business if RNIs are to be met.

The intake of visible fats (cooking oils) was not the only matter of concern. Hidden fats from meat products are another challenge. Pork alone provided about 25.8 g fat constituting 24.2% of total fat and 9.9% of total energy consumed. There is no doubt that meat is a great source of protein, but what accompanies the protein must also be considered. Pork provides about 13 g protein per 100 g, but also 37 g fat. Beef, chicken, or fish, on the other hand, provide about 20 g, 12 g, or 17 g of protein but only 4 g, 9 g, or 5 g fat per 100 g, respectively [39,40]. Pork, however, enjoys the highest popularity among the Chinese (Table 4), which points to another key issue to be tackled. To conclude, it would be desirable to complement the predominantly pork-based diet by an increased consumption of fish or more lean meats such as beef and chicken. Apart from the nutrient-density of foods, the biological quality of nutrients and their bioavailability must also be considered [52].

#### 4.3.2. Improving the Intake of Micronutrients in Rural and Urban Area

Pregnant Chinese women have been reported to be deficient in Fe, Zn, and Ca [12,54]. The 2002 NNHS affirmed inadequate dietary intake of Zn and Ca [50]. Data provided by the Deyang study shows substantial deficiencies in all minerals, with most average intakes falling short of the national RNI with a 20% allowance as a criterion (Table 3). According to the 2002 NNHS, the Chinese still consume a largely plant-based diet providing at least 50% of dietary energy and nutrients. Similarly, in the Deyang study, energy and nutrients were largely of plant origin. The high level of phytates in these foods decreases the bioavailability of critical nutrients by forming insoluble and indigestible complexes. The magnitude of this problem becomes especially clear when taking into account the fact that Fe deficiency was still found to be prevalent, despite the high total Fe intake reported among the Chinese population [58]. Hence, both insufficient intake as well as poor bioavailability might account for mineral deficiencies in China.

In our study, 26.2% of all women self-reported having suffered from slight or more severe forms of anemia before conceiving, equal to 19.3% and 38.8% in urban and rural areas, respectively. During pregnancy this percentage rose to 35.6% for the total sample, and accounted for 32.5% in the urban and 41.2% in the rural areas. Similarly, widespread prevalence of Ca deficiency has been self-reported before (13.1%) and during pregnancy (26.6%, 30.7% in urban *vs.* 19.0% in rural areas).

Bearing in mind that milk and dairy products provide the richest and most easily absorbed dietary sources of Ca, it might be advisable to further increase overall milk consumption. However, considering the greatly increased production of dairy products from slightly more than 10 million metric tons in 2001 to almost 39 million metric tons in 2009, consistent with an annual growth rate of 26% [59], such a casual recommendation has to be scrutinized as the growing demand for livestock products is likely to have an undesirable impact on the environment [19]. Hence, a moderate consumption (250 mL/day) of milk or dairy products (sample average 159 mL/day) is recommended, with special emphasis on the rural area. Shrimp is also another good source of Ca [53]. Furthermore, nuts (almonds), sunflower or sesame seeds, laver (seaweed) as well as green leafy vegetables are rich in Ca, though inferior due to a high fat content and/or low bioavailability [39,40]. Nevertheless, an increase in their consumption would help to fill the nutrient gap.

Tofu dishes or secondary tofu products were consumed by a relatively small proportion of urban (9.6%) and rural (7.5%) subjects. The following provides a basis for placing greater emphasis on the consumption of tofu in the diet. As “tofu may be the most popular food made of soy and is an inexpensive, nutritious, and versatile food” [60], it is natural to pursue the approach of improving Ca intake first and foremost by boosting the popularity of tofu. Therefore, its supposed equivalence to dairy products as a source of Ca and its superiority in terms of ethical principles (by not exploiting animals for their secondary products) should be the focus. Soybean curd is greatly enriched in Ca as the traditional processing method includes the addition of different coagulants usually powdered gypsum (Ca sulfate) [60]. Distressingly, study results by Ma indicate that phytates in soy products inhibit the absorption of Ca, Fe, and Zn [58]. By contrast, investigations by Weaver *et al.* [61] maintain that the efficiency of Ca absorption from tofu is equivalent to that of cow’s milk. Furthermore, among the few Ca-fortified foods, tofu provides Ca in concentrations comparable to milk in an acceptably sized portion. In addition, both cow’s milk and tofu provide good quality protein [61], with soy-protein being the only plant protein approaching the quality of animal protein [53]. Since 1997, the Chinese government has urged an increase in milk production and consumption as an effective approach to improve the Ca intake of the Chinese population [62,63]. Integrating all this core information, the emphasis on improving Ca intake must not be restricted to dairy products, but rather should be extended to include selected Ca-fortified soybean products such as tofu.

In this study, the overall Fe and Zn intake met only 53.4% and 67.9% of the RNI. Taken together with the above-mentioned impaired bioavailability in plant-based diets, this is quite alarming. The estimated absorption rates are 15%–35% and 2.8% for heme and non-heme Fe, respectively [49]. Therefore, the consumption of heme Fe (meat and fish) together with enhancers of Fe absorption should be encouraged, since heme Fe is much more easily absorbed than non-heme Fe (most dietary iron), the absorption of which greatly depends on the balance between inhibitors and promoters. Liver is naturally low in fat and is a valuable source of micronutrients such as Zn and vitamins A, B, and D, as well as Fe [53]. Official recommendations dissuade pregnant women in the first and second trimesters as well as those who wish to become pregnant from consuming liver due to assumed teratogenicity for the fetus [56]. However, as there is no reason to refrain from moderate consumption of liver (20–50 g) during the third trimester, one portion every two weeks is recommended. In any case, even small amounts of meat, complementing a largely vegetable-based dish, can boost the availability of non-heme Fe, as the “meat factor” has a positive effect on non-heme Fe absorption. This is gratifying when considering that Chinese lunch and dinner are commonly accompanied by at least some meat. However, the strongest promoter of non-heme Fe absorption is vitamin C from fresh fruits or vegetables. As the interaction takes place in the gastrointestinal tract, positive effects on non-heme Fe absorption are the greatest when promoters are consumed in the same meal. Hence, as an accompanying measure, the consumption of a glass of freshly pressed orange juice or a whole orange is recommended to improve the Fe absorption from plant foods. Vegetables high in vitamin C (bitter gourd, cauliflower, sweet peppers) or additionally in citric, malic and tartaric acid, such as potatoes, tomatoes, or cabbage are good alternatives for improving Fe absorption. A range of traditional food preparation practices enhances the Fe absorption by reducing the levels of phytates in plant foods (fermentation, germination, milling, soaking and roasting) [53]. According to local food culture and the result of NutriSurvey, sesame seeds, soybean milk film, pumpkin seeds, and shitake mushrooms are recommended as Fe sources for both rural and urban women. Abalone (expensive seafood) could also be a good example of food rich in Fe.

Good sources of Zn include shitake mushrooms, sesame seeds, sunflower seeds, pumpkin seeds, red meat, liver, seafood, eggs, and dairy products. Grains and legume seeds also provide considerable amounts of Zn. However, efficiency of absorption is inversely related to the content of phytates [53].

### 4.4. Limitations of This Study

Due to lack of published evidence, food consumption patterns of the Deyang study were compared with the NNHS, which included a small sample from Sichuan province and a smaller sample of pregnant women in third trimester. This was the best possible comparison for the time being.

Dietary assessment is based on self-reported data that tend to under-or overestimate actual food consumption due to several reasons [64,65,66,67,68,69]. The pattern of sharing a meal and complexity of Chinese dishes make an accurate recall of the types and amounts of foods consumed more difficult. Another challenge of dietary assessment relates to the variability of food composition databases, and the need “to set global micronutrient recommendations based on ‘harmonizing’ those from IOM, WHO/FAO, UK” [70] including Chinese DRIs adopted by INFSC 2002/2004.

Furthermore, a study design based on the single 24-h recall method is not suitable for estimating the proportion of the population that consumes adequate or inadequate diets, since a single 24-h recall is not representative of the long-term, usual intake of *individuals* due to high day-to-day variability [65,68,69,71]. However, the mean of one-day intakes by individuals drawn from a large, representative sample is not affected by day-to-day variations, thus the use of a single 24-h recall—representing an appropriate mix of the days of the week—was a good estimate of the *group’s usual mean* intake [72]. Seasonal variations are not reflected in our study due to the cross sectional study design.

Research shows substantial variations in the amount of cooking oil added during food preparation across time, regional differences, and socio-economic status [73]; this variation discourages a definition for recipe standards. However, as the estimate of cooking oil consumption by every individual may lead to substantial under- or overestimation of fat intake, the generation of standard recipes is appropriate. Secondly, as we relied on recipe files, we could not address the problem of oil residues in cooking utensils as a major error source. This approach is substantiated when comparing the average oil amount consumed per capita day (46 g) with amounts reported in the 2002 NNHS including individuals of all ages (41 g) [20].

Another limitation is the fact that despite the random selection of medical institutions the results cannot be generalized for the total population of pregnant women in the study area. However, the percentage of the population covered by health insurance schemes in China increased from 22.1% in 2003 to 87.1% in 2008. In terms of rural areas, the coverage by the rural cooperative medical scheme (RCMS), which is heavily subsidized by national and local government, increased from 12.7% to 94.2% from 2003 to 2009 [74]. Between 2006 and 2010 the majority of Chinese women (96%) delivered in medical institutions [26]. Based on this additional information it can be assumed that pregnant women recruited from the hospitals constitute a good representation of the population of pregnant women in the study area.

## 5. Conclusions

Before conception, the prevalence of a low BMI was more pronounced in rural areas as compared to urban areas. Urban pregnant women had more desirable intakes in all nutrients (except for fat) than rural women but still had low intakes of certain micronutrients. A more balanced diet for pregnant women in the Deyang region could be achieved by serving non-fried rather than fried dishes, the partial substitution of lean meats for high-fat meats, increased consumption of seafood, more regular consumption of animal organs (in particular, liver), sufficient consumption of eggs, a more privileged position of tofu, whole grains, colorful fruits and vegetables, and low-fat dairy products in the daily diet. Adequate amounts of pulses, selected seeds or nuts make the balanced diet complete. To conclude, in compliance with Ma [58], dietary diversification is suggested as the key intervention to improve the macro- and micro-nutrient intake of pregnant women living in Sichuan province. Succinctly, women are advised to limit cooking oil consumption as a major source of dietary fat, along with increasing nutrient-dense foods.

The great variety of culturally acceptable food choices is a decisive advantage in creating a diet that best fulfills the nutrient requirements of pregnant Chinese women. Most desirable would be improving nutritional status even before conceiving. Adherence to at least some of the recommendations given, with particular emphasis on nutrition education especially for pregnant women from rural areas, can narrow the nutrient gap considerably.
